# Furaldehyde substrate specificity and kinetics of *Saccharomyces cerevisiae* alcohol dehydrogenase 1 variants

**DOI:** 10.1186/s12934-014-0112-5

**Published:** 2014-08-09

**Authors:** Boaz Laadan, Valeria Wallace-Salinas, Åsa Janfalk Carlsson, João RM Almeida, Peter Rådström, Marie F Gorwa-Grauslund

**Affiliations:** Applied Microbiology, Department of Chemistry, Lund University, P.O. Box 124, SE-22100 Lund, Sweden; Department of Chemistry-BMC, Uppsala University, Box 576, SE-751 23 Uppsala, Sweden; Embrapa Agroenergy, Parque Estação Biológica, PqEB, W3 Norte (Final), 70770-901 Brasília, DF Brazil

**Keywords:** Alcohol dehydrogenase, NADH, Site-directed mutagenesis, Furfural, HMF

## Abstract

**Background:**

A previously discovered mutant of *Saccharomyces cerevisiae* alcohol dehydrogenase 1 (Adh1p) was shown to enable a unique NADH-dependent reduction of 5-hydroxymethylfurfural (HMF), a well-known inhibitor of yeast fermentation. In the present study, site-directed mutagenesis of both native and mutated *ADH1* genes was performed in order to identify the key amino acids involved in this substrate shift, resulting in Adh1p-variants with different substrate specificities.

**Results:**

*In vitro* activities of the Adh1p-variants using two furaldehydes, HMF and furfural, revealed that HMF reduction ability could be acquired after a single amino acid substitution (Y295C). The highest activity, however, was reached with the double mutation S110P Y295C. Kinetic characterization with both aldehydes and the *in vivo* primary substrate acetaldehyde also enabled to correlate the alterations in substrate affinity with the different amino acid substitutions.

**Conclusions:**

We demonstrated the key role of Y295C mutation in HMF reduction by Adh1p. We generated and kinetically characterized a group of protein variants using two furaldehyde compounds of industrial relevance. Also, we showed that there is a threshold after which higher *in vitro* HMF reduction activities do not correlate any more with faster *in vivo* rates of HMF conversion, indicating other cell limitations in the conversion of HMF.

## Background

Cost-efficient bioethanol production from lignocellulosic hydrolysates is in demand nowadays as a renewable liquid fuel [1,2]. However, the efficient fermentation of the complex raw material confronts several challenges, one of which being growth-inhibiting substances released during pretreatment and hydrolysis of lignocellulose [3]. During pretreatment of the biomass, two furaldehydes, HMF (5-hydroxymethylfurfural) and 2-furfural, are produced as the result of dehydration of hexose and pentose sugars, respectively [4,5]. Both HMF and furfural inhibit growth of *S. cerevisiae* and show synergistic effects [6]. Other reported effects include the decrease in ethanol yield and productivity [7] and the inhibition of several key enzymes by furfural [8], among which is alcohol dehydrogenase (EC 1.1.1.1). Native *S. cerevisiae* cells are able to reduce HMF and furfural *in vivo* to the less inhibitory alcohols [6,9], however at a low and strain-dependent rate.

We previously isolated and characterized a mutated gene variant of *S. cerevisiae ADH1* [10] from strain TMB3000, found at a spent sulfite liquor fermentation plant [11]. Whereas native Adh1p (natAdh1p) primarily catalyzes the reduction of acetaldehyde to ethanol using NADH as a co-factor [12], and was shown to reduce the smaller molecule formaldehyde [13], the mutated variant (mutAdh1p) also reduced the larger compound HMF using NADH as a co-factor [10]. All other known *S. cerevisiae* HMF reductases, including Adh6p, had been described as being NADPH dependent [14]. The nucleotide sequence of mutAdh1p revealed six amino acid substitutions [10]. While three of the reported mutations were present in previously published sequences [15] the other three mutations were novel and shown to reside in the vicinity of the substrate-binding pocket [10].

**Figure 1:**
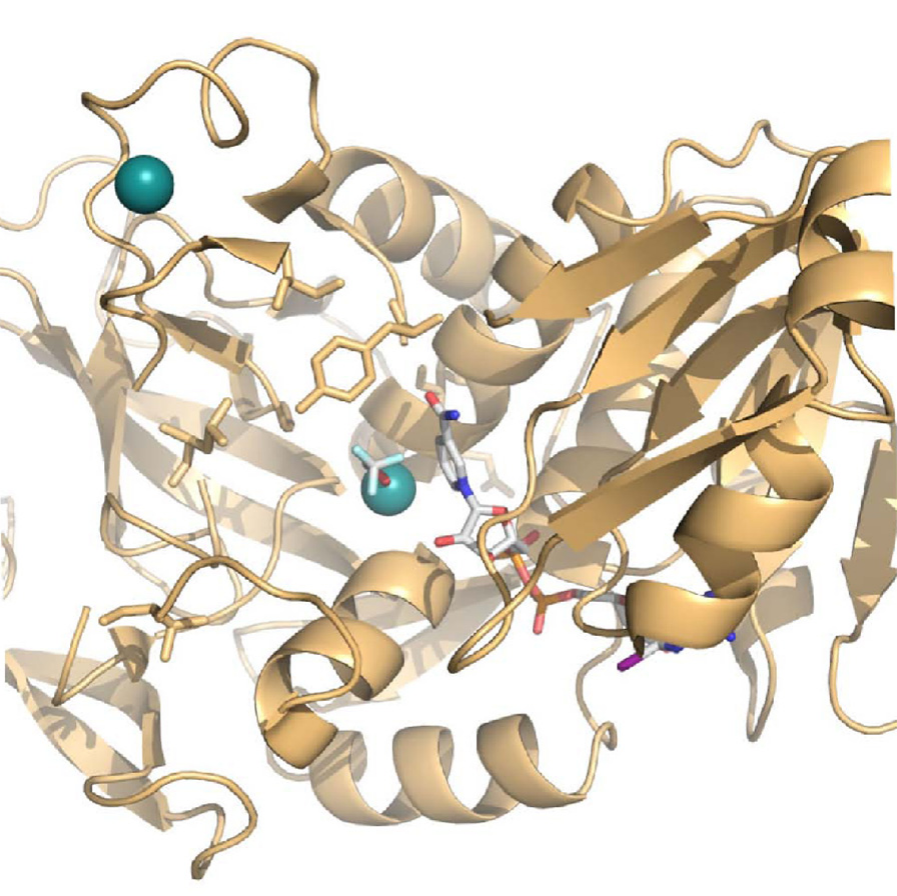
**Active site of wild-type Adh1p from** ***S. cerevisiae*** **.** Residues mutated during this work (Val59, Ser110, Leu117, Gln148, Ile152 and Tyr295) are presented as stick models (orange) together with NAD(H) analogue nicotinamide-8-iodo-adenine-dinucleotide and substrate analogue trifluoroethanol (white). Structural and active-site zink are shown as spheres (green). The image was created in PyMol (www.pymol.org) from the atomic coordinates in 2HCY.

In the present study, the effect of individual mutations was evaluated through site-directed mutagenesis of both natAdh1p (Figure 1) and mutAdh1p. The different variants displayed a wide range of reduction rates with several substrates and the comparison revealed the effects of both novel and previously reported mutations. Five variants were kinetically characterized, revealing distinct differences between affinity constants and maximal velocity values for both furaldehyde and acetaldehyde substrates. The variant displaying the highest *in vitro* activity with HMF was evaluated for *in vivo* HMF reduction.

## Results

### Generation and *in vitro* activity of Adh1p-variants

In the present study we generated a group of Adh1p-variants based on six mutations previously identified in the industrial *S. cerevisiae* strain TMB3000. Through enzymatic assays using the furaldehyde compounds HMF and furfural, alterations in the reduction capacity of the variants were analyzed in relation to the amino acid changes.

The first three variants were created by mutagenesis of the originally isolated mutated *ADH1* gene from TMB3000 [10] encoding a protein designated here as mutAdh1p (Table 1). The three novel mutations found within the protein (S110P, L117S, Y295C) were changed individually *back* to their native state. The resulting protein variants were designated, respectively, mutAdh1p-rev110, mutAdh1p-rev117 and mutAdh1p-rev295 (Table 1).

**Table 1:** **Amino acid changes among the different Adh1p-variants**

**Variant**	**Position**					
	**59**	**110**	**117**	**148**	**152**	**295**
natAdh1p	V	S	L	Q	I	Y
mutAdh1p	T	P	S	E	V	C
mutAdh1p-rev110	T	S	S	E	V	C
mutAdh1p-rev117	T	P	L	E	V	C
mutAdh1p-rev295	T	P	S	E	V	Y
natAdh1p-m110	V	P	L	Q	I	Y
natAdh1p-m295	V	S	L	Q	I	C
natAdh1p-m110, 295	V	P	L	Q	I	C
mutAdh1p-rev110,117	T	S	L	E	V	C

**Figure 2:**
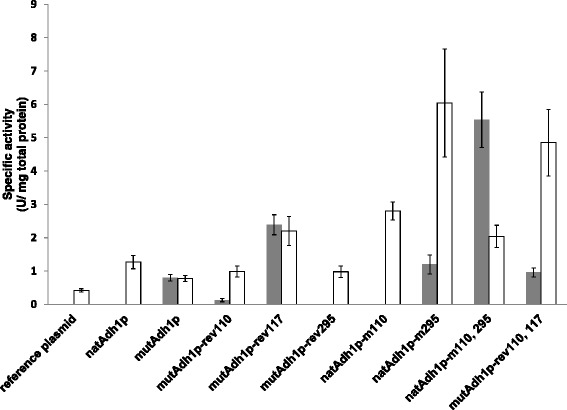
**Specific activity against HMF (filled bars) and furfural (empty bars) of strains carrying different variants of Adh1p (see Table** 1 **for description).** Absence of bar means not activity could be detected. One unit (U) corresponds to 1 μmol of NADH oxidized per min, at 30°C and pH 6.7.

NADH-dependent reduction of HMF and furfural were compared using the lysate of the strains carrying the three new Adh1p-variants, the strain carrying mutAdh1p and a control strain (reference plasmid) (Figure 2). mutAdh1p exhibited an HMF activity of 0.80 U/mg total protein, while cell extract of the reference strain showed no activity with HMF. The three mutants, however, showed a significant change of activity with altering a single amino acid. Reversion at position 110 (mutAdh1p-rev110) led to over 80% reduction in HMF activity (0.13 U/mg total protein), while reversion at position 295 (mutAdh1p-rev295) abolished HMF activity, indicating a potential role in the acquired HMF reduction ability. In contrast, reversion at position 117 (mutAdh1p-rev117) led to a 3-fold increase (2.39 U/mg total protein) in specific activity against HMF (Figure 2).

With furfural, mutAdh1p showed an activity of 0.78 U/mg total protein while the control strain showed a decrease of over 40% to 0.42 U/mg total protein (Figure 2). Each reversion led to an increase in furfural reduction: more than 20% (0.99 U/mg total protein and 0.98 U/mg total protein) for mutAdh1p-rev110 and mutAdh1-rev295, respectively and almost a 3-fold increase in activity for mutAdh1p-rev117 (2.20 U/mg total protein).

In view of these results, natAdh1p was mutated at positions 110 and 295. Native *ADH1* gene was amplified from genomic DNA of CEN.PK 113-5D and overexpressed in the same strain (designated CEN.PK 113-5D [natAdh1p]). Site-directed mutagenesis of native *ADH1* resulted in two more protein variants designated natAdh1p-m110 (S110P) and natAdh1p-m295 (Y295C) (Table 1). NADH-dependent HMF and furfural activities were determined for the newly constructed strains (Figure 2). As expected from previous results, natAdh1p showed no activity against HMF. natAdh1p-m110 did not show activity with HMF either. natAdh1p-m295, on the contrary, showed an activity of 1.20 U/mg total protein, which represents a 50% increase compared with mutAdh1p. With furfural, all variants displayed a considerable increase in activity as compared with mutAdh1p: over 60% for natAdh1p (1.27 U/mg total protein) as well as 3.6-fold (2.80 U/mg total protein) and 7.7-fold (6.04 U/mg total protein) increase for natAdh1p-m110 and natAdh1p-m295, respectively (Figure 2).

In order to further understand how different amino acids influenced the substrate specificity of Adh1p, two more mutants were created. NatAdh1p was mutated at positions S110P and Y295C, and named natAdh1-m110, 295 (Table 1). In mutAdh1p two changes, P110S and S117L were performed, resulting in variant mutAdh1p-rev110, 117. Reversion at positions 110 and 117 gave slightly higher HMF activity (0.96 U/mg total protein) than mutAdh1p but the highest HMF activity of all variants was obtained for natAdh1p-m110, 295 (5.54 U/mg total protein) (Figure 2). Furfural activity was high for both new variants, with 4.85 U/mg total protein for mutAdh1p-rev110, 117 and 2.04 U/mg total protein for natAdh1-m110, 295 (Figure 2).

The possible effect of *ADH1* gene dosage on Adh1p activities was determined using a relative quantification assessment of plasmid copy number between strains carrying mutAdh1p and natAdh1p-m110, 295. qPCR data showed indistinguishable quantification values between both strains (data not shown). Therefore, since no difference in plasmid copy number was observed between these strains at the moment of harvesting, the differences in activities previously described with both furaldehydes are rather a direct result of the mutations in the *ADH1* gene. In addition, and considering that all strains were harvested at comparable physiological state and cell density, it is very likely that the amino acid substitutions, rather that differences in gene dosage, are also responsible for the variations in activities observed between the other strains.

### Kinetics of Adh1p-variants

Variants natAdh1p, mutAdh1p and its derivative that was reverted at position 117 (mutAdh1p-rev117), as well as natAdh1p-m110, 295 and mutAdh1p-rev110, 117 were further kinetically characterized. Kinetic parameters were determined by introducing the corresponding genes in strain BY4741 that lacked *ADH1* gene (Table 2) and measuring NADH-dependent HMF, furfural and acetaldehyde reduction in the cell extracts. The parameters Vmax, Km and Ki were determined from the model described in the section Materials and methods; where the parameter K_i_ represents the inhibition factor, with higher values implying lower inhibition. [S]_Vmax_ represents the substrate concentration at which the experimental Vmax (observed V_max_) was measured. The RSQ value stands for coefficient of determination *R*^*2*^, describing the variability between the data set and the statistical model, with value 1 specifying a perfect correlation. The background activity in cell extract of the ΔAdh1 strain was low for all the substrates (0.02 U/mg total protein for acetaldehyde and furfural; 0.01 U/mg total protein for HMF).

**Table 2:** **Strains used in the study**

***S. cerevisiae*** **strains**	**Relevant genotype**	**Reference**
CEN.PK 113-5D	*Mat*a *ura3*	[16]
Control (reference plasmid)	CEN.PK 113-5D, YEplacHXT	[10]
CEN.PK 113-5D [natAdh1p]	CEN.PK 113-5D, YEplacHXT-natADH1	This study
CEN.PK 113-5D [mutAdh1p]	CEN.PK 113-5D, YEplacHXT-mutADH1	[10]
CEN.PK 113-5D [mutAdh1p-rev110]	CEN.PK 113-5D, YEplacHXT-mutADH1-rev110	This study
CEN.PK 113-5D [mutAdh1p-rev117]	CEN.PK 113-5D, YEplacHXT-mutADH1-rev117	This study
CEN.PK 113-5D [mutAdh1p-rev295]	CEN.PK 113-5D, YEplacHXT-mutADH1-rev295	This study
CEN.PK 113-5D [natAdh1p-m110]	CEN.PK 113-5D, YEplacHXT-natADH1-m110	This study
CEN.PK 113-5D [natAdh1p-m295]	CEN.PK 113-5D, YEplacHXT-natADH1-m295	This study
CEN.PK 113-5D [natAdh1p-m110, 295]	CEN.PK 113-5D, YEplacHXT-natADH1-m110, 295	This study
CEN.PK 113-5D [mutAdh1p-rev110, 117]	CEN.PK 113-5D, YEplacHXT-mutADH1-rev110, 117	This study
BY4741	Mat-a his3Δ1 leu2Δ0 met15Δ0 ura3Δ0, deleted ORF YOL086C (adh1)	EUROSCARF collection, Heidelberg, Germany
BY4741 [natAdh1p]	BY4741, YEplacHXT-natADH1	This study
BY4741 [mutAdh1p]	BY4741, YEplacHXT-mutADH1	This study
BY4741 [mutAdh1p-rev117]	BY4741, YEplacHXT-mutADH1-rev117	This study
BY4741 [natAdh1p-m110,295]	BY4741, YEplacHXT-natADH1-m110, 295	This study
BY4741 [mutAdh1p-rev110,117]	BY4741, YEplacHXT-mutADH1-rev110, 117	This study

The cell cultures of the different BY4741 strains used for kinetic characterization were harvested at the same physiological state and comparable cell densities, and the analysis focused on the differences in activities (with HMF and furfural) within each variant in relation to the natural substrate acetaldehyde.

The results from the kinetic characterization showed that the various mutations altered the activity of the variants with the substrates to different extent (Table 3). For natAdh1p, HMF activity was not detected, while the activity with furfural was about 14% of that measured with acetaldehyde. The variant mutAdh1p, on the other hand, showed activity with HMF, corresponding to about 66% of the activity obtained with acetaldehyde as substrate. For this variant, the activity with furfural was lower than that with HMF, since it was only around 34% of that obtained with acetaldehyde (Table 3). The other variant studied, mutAdh1p-rev117 showed a different preference in substrate. This variant displayed, with HMF and furfural respectively, around 27% and 50% of the activity obtained with acetaldehyde (Table 3). The variant natAdh1p-m110, 295 showed similar ratios on activities as the mutAdh1p, that is, its activity with HMF was close to 70% of the activity measured with acetaldehyde, and with furfural only around 34% of that obtained with the reference substrate. Finally, mutAdh1p-rev110, 117 showed a low activity with HMF and furfural (11% and 24% of the activity obtained with acetaldehyde respectively) (Table 3).

**Table 3:** **Ratio of activities for the Adh1p-variants with substrates HMF and furfural in relation to the activity obtained with the natural substrate acetaldehyde during the kinetic characterization**

**Ratio of activities**	**natAdh1p**	**mutAdh1p**	**mutAdh1p-rev117**	**natAdh1p-m110, 295**	**mutAdh1p-rev110, 117**
$\frac{Vmax\ HMF}{Vmax\ acetaldehyde}$	0.00	0.66	0.27	0.67	0.11
$\frac{Vmax\ furfural}{Vmax\ acetaldehyde}$	0.14	0.34	0.51	0.34	0.24

The kinetics also highlighted differences in affinity constant (Km) and substrate inhibition constants (Ki) between the substrates (Table 4). For natAdh1p, the affinity constant K_m_ was much lower for acetaldehyde than for furfural (0.22 mM vs. 18.80 mM). However, this variant did not show inhibition by furfural at concentrations up to 30 mM, while inhibition by acetaldehyde was observed around 1 mM (Table 4). The K_m_ values obtained for mutAdh1p were 1.69 mM (acetaldehyde), 4.30 mM (HMF) and 0.04 mM (furfural) (Table 4). Inhibition with acetaldehyde and HMF was observed around concentration of 10 mM of these substrates, while with furfural inhibition was observed at lower concentrations (around 0.25 mM). For the variant mutAdh1p-rev117, the K_m_ values obtained were 1.74 mM, 4.28 mM and 0.33 mM for acetaldehyde, HMF and furfural respectively (Table 4). Inhibition by acetaldehyde was observed above 25 mM, for HMF around 10 mM while with furfural above 1 mM. For natAdh1p-m110, 295, the K_m_ values were lower than one for acetaldehyde (0.83 mM) and furfural (0.13 mM), and with HMF the K_m_ calculated was 9.35 mM. This variant was less inhibited by HMF (above 20 mM) than by acetaldehyde (inhibition observed above 5 mM). With furfural, however, inhibition was observed at concentrations above 0.50 mM. Finally, the Km values obtained for mutAdh1p-rev110, 117 were 3.03 mM, 13.10 mM and 0.34 mM for acetaldehyde, HMF and furfural respectively. Inhibition by acetaldehyde was observed at concentrations above 10 mM, while inhibition by HMF and furfural were evident above 20 mM and 1 mM respectively (Table 4).

**Table 4:** **Kinetic parameters of the Adh1p-variants with acetaldehyde, HMF and furfural as substrates**

**Variant**	**Substrates**	**Vmax (mU/mg)**	**Km (mM)**	**Ki (mM)**	**[S]**_**Vmax**_ **, (obsV**_**max**_ )*	**RSQ**
natAdh1p	*Acetaldehyde*	45070	0.22	12.53	1 (35300)	0.987
	*HMF*	Not detected				
	*Furfural*	6530	18.8	1363	30 (4345)	0.970
mutAdh1p	*Acetaldehyde*	4736	1.69	49	10 (3700)	0.968
	*HMF*	3120	4.30	9.82	5–10 (1320)	0.984
	*Furfural*	1625	0.04	1.07	0.25 (1230)	0.974
mutAdh1p-rev117	*Acetaldehyde*	23060	1.74	96	10–25 (17600)	0.979
	*HMF*	6320**	4.28	n.d	10 (4900)	0.975
	*Furfural*	11700	0.33	4.40	1 (7600)	0.992
natAdh1p-m110, 295	*Acetaldehyde*	16509	0.83	46	5 (14300)	0.970
	*HMF*	11090	9.45	20.80	10–20 (4560)	0.999
	*Furfural*	5657	0.13	1.32	0.25–0.5 (3480)	0.984
mutAdh1p-rev110,117	*Acetaldehyde*	32455	3.03	56	10 (22000)	0.974
	*HMF*	3640	13.10	27.30	20 (1550)	0.980
	*Furfural*	7792	0.34	6.66	1 (5320)	0.986

*[S]_Vmax_ – substrate concentration (mM) at observed V_max_ (obsV_max_, mU/mg), **V_max_ and K_m_ values derived from a model *without* substrate inhibition factor, see text for details.

### *In vivo* HMF conversion

Anaerobic batch fermentations in the presence of 2 g/L HMF were carried out to compare the *in vivo* HMF reduction capacity of strain CEN.PK 113 5D [mutAdh1p] and the strain showing the highest *in vitro* HMF/acetaldehyde reduction ratio CEN.PK 113 5D [natAdh1p-mut110, 295]. A control strain carrying a reference plasmid was also included. The concentration of HMF was chosen according to levels previously reported in spruce hydrolysate [17].

**Figure 3:**
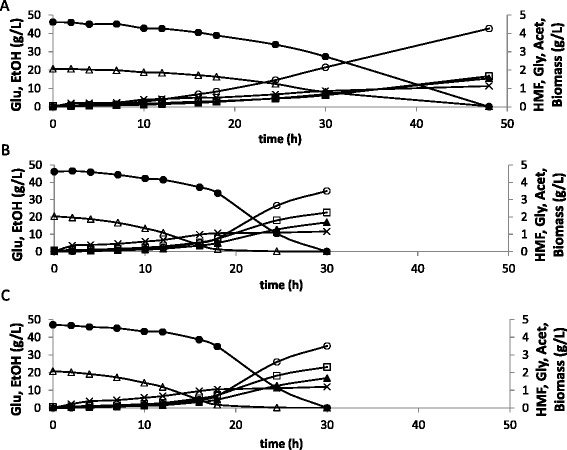
**Comparison of glucose/HMF consumption and acetate/glycerol/ethanol/biomass formation during anaerobic cultivations in the presence of HMF for the control strain (A) and strains CEN.PK 113 5D [mutAdh1p] (B) and CEN.PK 113 5D [natAdh1p-mut110, 295] (C).** Legend: ●-glucose, ○-glycerol, X-acetate, ▲-ethanol, ∆-HMF, □-biomass. The experiment was performed in biological duplicates and the figure shows the data of one representative profile for each strain. The standard deviation between replicates was less than 15%.

The *in vivo* HMF uptake rate of the control strain (0.14 g/g cell. h) was the same as the one previously reported for the *S. cerevisiae* strain CBS 8066 under similar conditions [6]. For the strains carrying the two mutants, the *in vivo* values were equivalent, and more than three times higher than for the control strain (Table 5). In agreement with this result, both strains overexpressing the mutated *ADH1* genes performed very similarly during the fermentations (Figure 3, Table 5) and displayed much higher glucose consumption rate than the control strain (Table 5). Strains overexpressing *ADH1* also showed slightly lower concentrations of glycerol when compared to the control. But final ethanol yields and product distribution was similar for the rest of metabolites between the three strains (Table 6). When the values of *in vitro* specific activity were compared with the *in vivo* rates of HMF uptake, it was found that the capacity for HMF reduction *in vitro* was around five times and thirty seven times higher (for CEN.PK 113-5D [mutAdh1p] and CEN.PK 113-5D [natAdh1p-m110, 295] respectively) than during the fermentations (Table 5).

**Table 5:** ***In vitro*** **and** ***in vivo*** **conversion rate of HMF and specific growth, glucose consumption and ethanol production rates in anaerobic batch fermentation in defined mineral medium supplemented with 2 g/l HMF**

**Strain**	***In vitro*** **HMF reduction** ^**a**^ **(g/g cells. h)**	***In vivo*** **conversion rate (g/g cells.h)**	**μ (1/h)**	**q**_**glucose**_ **(g/g cells. h)**	**q**_**ethanol**_ **(g/g cells. h)**
Control	nd^b^	0.14 ± 0.00	0.07 ± 0.00	1.54 ± 0.09	0.58 ± 0.06
CEN.PK 113-5D [mutAdh1p]	2.57 ± 0.32	0.47 ± 0.03	0.13 ± 0.01	2.96 ± 0.63	1.19 ± 0.09
CEN.PK 113-5D [natAdh1p-m110, 295]	17.81 ± 2.68	0.48 ± 0.00	0.13 ± 0.01	3.08 ± 0.70	1.17 ± 0.16

^a^The *in vitro* activities for these strains were recalculated as gHMF converted per gram of cells per hour. This allowed the comparison between the conversion rates during the fermentation and the *in vitro* measurements. ^b^Not detected.

Rates were calculated during exponential growth. Presented values are the mean of two independent biological replicates.

**Table 6:** **Ethanol, biomass, glycerol and acetate yields in anaerobic batch fermentation in defined mineral medium with 40 g/l glucose and supplemented with 2 g/l HMF**

**Strain**	**Y**_**ethanol**_	**Y**_**biomass**_	**Y**_**glycerol**_	**Y**_**acetate**_
Control	0.38 ± 0.06	0.04 ± 0.01	0.10 ± 0.01	0.03 ± 0.00
CEN.PK 113-5D [Adh1 (59, 110, 117, 148, 152, 295)]	0.41 ± 0.06	0.05 ± 0.01	0.09 ± 0.02	0.03 ± 0.01
CEN.PK 113-5D [Adh1 (110, 295)]	0.38 ± 0.04	0.05 ± 0.01	0.08 ± 0.01	0.03 ± 0.00

Yields are expressed as g of product per g of glucose. Presented values are the mean of two independent biological replicates ± standard deviation.

## Discussion

The study aimed at unraveling the importance of the different mutations identified in a previously described Adh1p mutant displaying an unusual NADH-dependent HMF reductase activity [10]. Here we demonstrated the key role of Y295C mutation in HMF reduction, but also the overall negative impact of L117S mutation on furaldehyde reduction. We previously hypothesized that the replacement of tyrosine with cysteine at position 295 resulted in the widening of the substrate binding pocket, by enabling the accommodation of the bulky HMF molecule [10]. However, natAdh1p can reduce the bulky furfural substrate, as displayed here and elsewhere [18]. We therefore speculate that acquisition of HMF and increase in furfural reduction capability by Y295C mutation relies not only on an enlarged binding pocket, but also in the differences in properties, such as the polarity of the side chain, between tyrosine and cysteine. We also demonstrated that the acquired mutation at position 117, whose neighbor amino acids belong to the substrate-binding pocket [19], was disadvantageous for furaldehyde reduction as the reduction capacity to both furaldehydes was increased when the polar amino acid serine was reverted back to the original hydrophobic amino acid leucine. Finally, mutation S110P could not by itself generate activity against HMF as demonstrated with natAdh1p-m110, but it proved to be important for HMF reduction since reversion of the mutation in mutAdh1p-rev110 led to significantly lower reduction activity. Also combining S110P and Y295C mutations (natAdh1p-m110, 295) led to a 4.6-fold increase in specific activity towards HMF. In contrast, this combination led to a 3-fold decrease in furfural reduction activity whereas both individual mutations gave higher furfural reduction levels than natAdh1p. Therefore the impact of mutation S110P appears to be dependent on the substrate and the presence of other surrounding mutations. It should be emphasized however, that is not possible to explain the changes in activities obtained among the variants solely from the changes in the primary structure of the proteins. Although some changes in the protein properties can be inferred by the differences in the properties of the amino acids that have been altered (e.g. polarity and charge), the final effect that such changes can have in the biological function of the enzyme requires a deeper analysis. The prediction of possible relevant changes in the tertiary structures of the protein (for example in the active site or co-factor binding site) based on the mutations here described goes beyond the aim of this study.

Kinetic measurements also revealed important additional features for the mutations. First it enabled the analysis of the influence of the three mutations previously reported in natAdh1p at positions 59, 148 and 152 [15]. For example, the activity of mutAdh1p-rev117 with HMF was only 27% of that with acetaldehyde, while for natAdh1p-m110, 295 the activity with HMF was close to 70% of the activity obtained with acetaldehyde. Considering that these variants have the same amino acids positions at 110, 117 and 295 but differ by amino acids at positions 59, 148 and 152, the relevance of these three mutations on the enzymes properties was proven. The kinetic characterization also gave insights on how the alterations in particular amino acids affected the inhibition properties of the enzymes by the three analyzed substrates. For instance, although mutAdh1p-rev110, 117showed the relatively highest activity with furfural, it was inhibited by this substrate at very low concentrations (~1 mM) suggesting that the use of this variant for detoxification of high-furfural containing hydrolysates (up to 20 mM; [3]) would probably be limited.

The use of yeast strains with enhanced levels of furaldehyde reductases has proven to be a successful strategy for minimizing the negative effects of different compounds present in lignocellulosic hydrolysates [17,20]. In general, higher *in vitro* conversion rates of HMF (and furfural) have been correlated with improved fermentation performances in the presence of these inhibitors [6,21]. In our study, the *in vivo* HMF specific uptake rate could indeed be increased from 0.14 g/g cell. h (control strain) to a maximum value of 0.48 g/g cell.h when overexpressing *ADH1-*variants. However, the laboratory strain expressing the most promising mutant, natAdh1p-m110, 295, did not show better in *vivo* performances than its counterpart expressing the original mutated gene during anaerobic fermentation on glucose in the presence of 2 g/l HMF, despite higher measured Vmax for natAdh1pn110, 295. Nilsson *et al*. [22] obtained similar results during fed-batch fermentation with HMF. In that case, the *in vivo* conversion rate equaled the total reduction capacity of the cells for a poorly performing strain whereas the total *in vitro* enzyme activity was much higher than the specific *in vivo* uptake rate of HMF for the industrial isolate TMB3000 that carries *ADH1* original mutant. Taken together, these results suggest that there may be a limit above which the specific uptake rate of HMF cannot be improved by further increasing the reduction capacity of the cells. The adaptation response of *S. cerevisiae* to HMF has been shown to involve different components including inhibitor detoxification, alteration of pathways for ATP production and co-factor regeneration, transportation of toxic metabolites and degradation of damaged proteins [23]. Transcriptome studies have shown that multidrug-transporters were up-regulated in the presence of furaldehydes [24], but there is not information available on how fast the reduced forms of the furaldehydes are transported outside the cells and on the level of inhibition caused by these compounds. The availability of cofactors inside the cells may also explain the limitation observed in the *in vivo* HMF conversion. In contrast to *in vitro* enzymatic assays, where the co-factors are added in excess and the reaction under study is “isolated” from other cells reactions, the intracellular concentrations of co-factors are carefully balanced and regulated by the many other reactions in which both pairs, NADH/NAD^+^ and NADPH/NADP^+^ participate [25]. It has recently been shown that when yeast cells were grown in chemostat in the presence of HMF and furfural, the levels of both NAD(P)H and NAD(P)^+^ were perturbed and a reduction in biosynthetic capacity was observed, due to the drainage of NADPH in the presence of the aldehydes [26]. In another study, the NADH-specific conversion of HMF led to reduced glycerol and increase biomass formation in a continuous cultivation with defined mineral medium [17], which could indicate a competition for NADH usage. So the co-factor used during furaldehyde reduction may impact the product distribution in favor of either NADH or NADPH consuming routes. And under the stress imposed by the presence of HMF, the cells overexpressing *ADH1* variants may be able to use the available NADH for reduction purposes only as long as the regeneration of this co-factor can take place and at a fast enough rate.

## Conclusions

The results presented here unravel the impact of single mutations on the substrate specificity of a key *S. cerevisiae* metabolic enzyme and identify tyrosine 295 as the key amino acid to mutate for getting HMF reduction capacity. From a more applied perspective, the results reaffirm that high reduction capacity is a relevant trait for furaldehyde bio-detoxification. Combination of this feature with further improvements in other type of mechanisms involved in inhibitor tolerance, will lead to the development of more robust strains for 2nd generation chemical production.

## Materials and methods

### Strains and cultivation conditions

*Saccharomyces cerevisiae* strains are summarized in Table 2. The strains were stored in 15% glycerol at −80°C. Cells from freshly streaked SD-ura plates [27] were used for pre-culture inoculation. *Escherichia coli* strain DH5α (Life Technologies, Rockville, MD, USA) that was used for cloning purposes was grown in Luria-Bertani medium [27] supplemented or not with ampicillin (50 μg/mL) and also stored in 15% glycerol at −80°C.

### Molecular biology methods

Competent *E. coli* cells were prepared using the Inoue method [28]. Transformants were selected on LB plates [27] containing 50 μg/mL ampicillin (ICN Biomedicals, Aurora, OH, USA). The lithium acetate method was used for transformation of *S. cerevisiae* [29]. Transformants were selected on SD-ura [27] plates. Restriction enzymes, T4-nucleotide ligase and shrimp alkaline phosphatase were purchased from Fermentas (Vilnius, Lithuania) whereas Pwo polymerase was purchased from Roche Diagnostics AB (Bromma, Sweden). All enzymes were used according to the manufacturers’ recommendations. PCR fragments were purified using the E.Z.N.A cycle-pure kit (Omega Bio-Tek, Doraville, GA, USA). Plasmids were prepared using the QIAprep Spin Miniprep Kit (Qiagen, Hilden, Germany). DNA sequencing was performed using the Abi-Prism BigDye cycle sequencing kit (Applied Biosystems, Weiterstadt, Germany). Plasmid YEplacHXT [17] was used for cloning steps, its constitutive truncated HXT promoter [30] governing the overexpression of ORFs. Plasmids used in this study are described in Table 7.

**Table 7:** **Plasmids used in the study**

**Plasmid**	**Construction background**	**Reference**
YEplacHXT (reference plasmid)	YEplac195, HXT7p-PGKt URA3	[31]
YEplacHXT-natADH1	YEplacHXT, native *ADH1*	This study
YEplacHXT-mutADH1	YEplacHXT, mutated *ADH1*	[10]
YEplacHXT-mutADH1-rev110	YEplacHXT, mutated *ADH1*, P110S	This study
YEplacHXT-mutADH1-rev117	YEplacHXT, mutated *ADH1*, S117L	This study
YEplacHXT-mutADH1-rev295	YEplacHXT, mutated *ADH1*, C295Y	This study
YEplacHXT-natADH1-m110	YEplacHXT, native *ADH1*, S110P	This study
YEplacHXT-natADH1-m295	YEplacHXT, native *ADH1*, Y295C	This study
YEplacHXT-natADH1-m110,295	YEplacHXT, native *ADH1*, Y295C, S110P	This study
YEplacHXT-mutADH1-rev110, 117	YEplacHXT, mutated *ADH1*, P110S, S117L	This study

### Native *ADH1* cloning

Extraction of genomic DNA from strain CEN.PK 113-5D was conducted using the Y-PER kit (Pierce, Rockford, IL, USA) according to the manufacturer’s recommendations. Amplification of native ADH1 from CEN.PK 113-5D genomic DNA was performed as previously described [10] using primers Sense *Bam*HI and Antisense *Bgl*II (Table 8). The single resulting amplicon was cleaved with *Bam*HI and *Bgl*II and used for ligation with previously double-cleaved and de-phosphorylated YEplacHXT plasmid.

**Table 8:** **Primers used for site-directed mutagenesis**

**Primer name**	**Sequence (5′-3′)**
P110S sense	GTGAATTGGGTAACGAATCCAACTGTCCTCACGC
P110S antisense	GCGTGAGGACAGTTGGATTCGTTACCCAATTCAC
S117L sense	CTGTCCTCACGCTGACTTGTCTGGTTACACCCAC
S117L antisense	GTGGGTGTAACCAGACAAGTCAGCGTGAGGACAG
C295Y sense	CTCCATTGTTGGTTCTTACGTCGGTAACAGAGCTG
C295Y antisense	CAGCTCTGTTACCGACGTAAGAACCAACAATGGAG
S110P sense	GGTAACGAACCCAACTGTC
S110P antisense	GACAGTTGGGTTCGTTACC
Y295C sense	TTGGTTCTTGCGTCGGTAAC
Y295C antisense	GTTACCGACGCAAGAACCAA
Sense *Bam*HI	GGGGGGATCCATGTCTATCCCAGAAACTC
Antisense *Bgl*II	CTTTAGATCTTTATTTAGAAGTGTCAACAACG

### Site-directed mutagenesis

For site directed mutagenesis, a two-step PCR was used for the generation of an ORF containing the correct nucleotide changes. Plasmids YEplacHXT-mutADH1 and YEplacHXT-natADH1 (Table 7) were used as PCR templates for reverse mutagenesis of mutated *ADH1* from TMB3000 and mutagenesis of native *ADH1* from CEN.PK 113-5D, respectively. Table 8 lists the primers used for PCR mediated mutagenesis. The first round combined Sense *Bam*HI and corresponding antisense primer and, a separate reaction with primers Antisense *Bgl*II and corresponding sense primer. The two resulting amplicons were used in the second reaction as template with primers Sense *Bam*HI and Antisense *Bgl*II. The resulting single amplicon was digested and ligated as described previously. All PCR reactions had an annealing step at 55°C, 1 minute elongation time and 30 cycles. All Adh1p-variants are designated by the protein of origin (natAdh1p or mutAdh1p) followed by an indication of the position where the mutation or reversion was introduced. Therefore, natAdh1p-m295 refers to natAdh1p after mutagenesis at position 295; and mutAdh1p-rev117 refers to the mutAdh1p [10] after reverse mutagenesis of position 117 (Table 1). Strain names of either CEN.PK 113-5D or BY4741 hosts include their Adh1p designation in parentheses (Table 2).

### Protein extraction

The cultures were grown until late exponential phase, and harvest at similar cellular densities. Cells were washed twice with double distilled water before being re-suspended in Y-PER detergent (Pierce), 1.7 ml/g cells. After gentle shaking at room temperature for 50 minutes the suspension was centrifuged for 20 minutes at 15,000 g. The lysate was collected for spectrophotometric assays. Protein concentration was determined with the Coomassie protein assay reagent (Pierce) in the Bradford assay according to the manufacturer’s recommendations.

### Alcohol dehydrogenase activity and kinetics

NADH-dependent aldehyde reduction was determined according to [32], using 200 μM NADH. All measurements were performed in biological duplicates and technical triplicates at 30°C in a U-2000 spectrophotometer (Hitachi, Tokyo, Japan). Reduction kinetics was determined for acetaldehyde (range 500 μM–100 mM), furfural (100 μM–20 mM) and HMF (500 μM–20 mM). For natAdh1p, furfural was used up to 40 mM. One unit (U) corresponds to 1 μmol of NADH oxidized per min, at 30°C and pH 6.7. All the enzymatic activities are reported as unit of activity per milligram of total protein (U/mg total protein).

### Kinetic modeling

Modeling was performed according to the Michaelis-Menten equation with the addition of a substrate inhibition constant:

$$ V=\frac{Vmax\ .\ \left[ S\right]}{Km+\left[ S\right] + \frac{{\left[ S\right]}^2}{Ki}} $$

where V is velocity; Vmax is maximal velocity; [S] is substrate concentration; Km is affinity constant; and Ki is substrate inhibition constant. Parameter value estimation was according to the least square method, using the solver function in Microsoft® Excel 2002. HMF activity parameters for strain BY4741 [mutAdh1p-rev117] were estimated without the substrate inhibition constant due to a clear discrepancy of the substrate inhibition model with actual data.

### Batch fermentation with HMF

Inoculum cultures were grown overnight at 30°C in 1 L cotton plugged shake-flasks with 100 mL of double concentrated defined mineral medium [33] supplemented with 40 g/L glucose and 50 mM potassium hydrogen phthalate buffer (adjusted to pH 5.5 with potassium hydroxide) The same medium was used in batch experiments, without the buffer and, when indicated, with 2 g/L HMF. The starting OD_620_ was 0.2. Fermentation was carried out in 800 ml medium at 30°C in 1.4 L Multifors bioreactor (Infors, Switzerland). Anaerobic conditions were maintained by continuously sparging 0.2 L/min N_2_ gas. The pH 5.5 was kept constant by addition of 3 M KOH. The stirring rate was 200 rpm.

### Analysis of fermentation data

Samples for metabolites and OD were regularly withdrawn from the reactor. For biomass determination, 5 mL culture was washed with distilled water and dried on Gelman filters (ø 47 mm Supor-450, 0.45 μm) in a microwave oven (350 W) for 8 minutes. The biomass concentration was correlated with OD_620_ by dry weight measurements. Glucose, ethanol, HMF, glycerol and acetic acid were analysed by high performance liquid chromatography (HPLC; Waters Corporation, MA, USA) on an Aminex HPX-87H column (Bio-Rad, CA, USA) at 65°C. The mobile phase was 5 mM sulphuric acid with a flow of 0.6 mL/min. All compounds were detected with a refractive index detector (Shimadzu, Tokyo, Japan).

The specific HMF uptake rates were calculated according to the formula:

$$ {\mathrm{q}}_{\mathrm{hmf}} = \upmu\ .\ \mathrm{m} $$

where:

μ = maximum specific growth rate (1/h)

m: slope obtained after plotting HMF concentration (gHMF/L) in the Yaxis vs. cdw (g cell/L) in the X-axis at each time point of the exponential phase.

## Abbreviations

HMF: 5-(hydroxymethyl)furfural
OD: Optical density
SD-ura: Synthetic defined without uracil
cdw: Cell dry weight
HPLC: High performance liquid chromatography
qPCR: Quantitative polymerase chain reaction
